# Purification and characterisation of intracellular alpha-galactosidases from *Acinetobacter* sp.

**DOI:** 10.1007/s13205-015-0290-9

**Published:** 2015-03-20

**Authors:** Sirisha E, Ravichandra Potumarthi, Naveen A, Lakshmi Narasu Mangamoori

**Affiliations:** 1 Centre for Biotechnology, Institute of Science and Technology, Jawaharlal Nehru Technological University Hyderabad, Hyderabad, Telangana India; 2 School of Agriculture, Food and Wine, The University of Adelaide, Waite Campus, Urbrae, SA 5064 Australia

**Keywords:** α-Galactosidases, Acinetobacter, Purification, Multiple forms

## Abstract

Two alpha-galactosidases (Ag-I & Ag-II) were purified from *Acinetobacter* sp. Both the enzymes were monomeric with pH optima of 7.0 and molecular weight of 65 kDa for Ag-I and 37 kDa for Ag-II. The temperature optima for Ag-I was between 50 and 60 °C and that of Ag-II was 40 °C. Both the enzymes were strongly inhibited by metal ions Ag^2+^ and Hg^+^, pCMB and SDS (1 %). The enzymes were found to be active on both natural and synthetic substrates. Artificial substrate, *p*NPGal, has shown more affinity to enzyme than natural substrate raffinose. The half-life (*t*_1/2_) of Ag-I varied from 1.85 h at 90 °C to 7.6 h at 70 °C.

## Introduction

In recent years, a substantial amount of interest has been generated on carbohydrate-cleaving enzymes due to their potential industrial and therapeutic applications. α-Galactosidases are enzymes that belong to family of glycosyl hydrolases. Based on aminoacid sequences, alpha-galactosidases are included in four different families—GH4, GH27, GH36 and GH57 (Naumoff 2001). Most of the known bacterial and eukaryotic alpha-galactosidases with confirmed activity belong to GH27 and GH36, which constitute a superfamily (clan GH D) (Naumoff 2004).

α-Galactosidases catalyse hydrolysis of terminal α-1-6 linked galactose residues from different galactooligosaccharides, glycosphingolipids and glycoproteins (Dey and Pridham 1972) and also show transgalactosylation (Kato et al. 1982) and hemagglutination activity (del Campillo and Shannon 1982). Due to its diverse catalytic activity, the enzyme is used in food processing industry for improvement of nutritional value of legumes and animal feeds by removing the flatulence-causing factors (Patil et al. 2010), in beet sugar industry for crystallisation of sugars (Yamane 1971) and α-galactosidase producing organisms as probiotic (Chen and Mustapha 2012; Liu et al. 2014). Recombinant alpha‐galactosidases such as Fabrazyme™ and Replagal™ are being currently employed in the treatment of Fabry’s disease which is a rare X‐linked lysosomal storage disorder (Tsuboi and Yamamoto 2012; Desnick 2004). The enzyme was reported to be applied for the conversion of Type ‘B’ erythrocytes to Type ‘O’ erythrocytes (Gao et al. 2011) and also in xenotransplantation (Zeyland et al. 2013). This enzyme is also used in bleaching of softwood in the paper industry (Clarke et al. 2000).

Although α-galactosidases were isolated and purified from various organisms (Singh and Kayastha 2012; Puchart et al. 2001; Saishin et al. 2010; Gao and Arthur 1999), the versatility of this enzyme and the demand for stable α-galactosidase are constantly increasing despite the availability. The present work was taken up to isolate and purify thermostable α-galactosidase from *Acinetobacter* sp., which will be later applied in degradation of galacto-oligosaccharides to increase nutritive value of legumes.

## Materials and methods

### Materials

Media constituents were acquired from HiMedia Laboratories, India. *ρ*-Nitrophenyl-a α-d-galactopyranoside (ρNPGal), Stachyose, Phenyl Methyl Sulphonyl Flouride (PMSF) and *k*-Carrageenan were procured from Sigma-Aldrich Inc, USA. Ion exchange matrix-Q-Sepharose fast flow and gel filtration matrix-Sephacryl S-300 were brought from GE Healthcare, USA.

### Microorganisms

Microorganisms showing α-galactosidase activity were isolated from soil collected from sugarcane industries situated at Sangareddy, Andhra Pradesh. The cultural, morphological and biochemical characteristics of the isolate were identified according to Bergey’s Manual of Determinative Bacteriology, 8th edition (Buchanan and Gibbons 1974) and confirmed by 16srRNA sequencing analysis.

### Crude enzyme preparation

A loopful of the isolate G1 was inoculated into optimised fermentation medium comprising raffinose 25 g/L, tryptone 10 g/L, K_2_HPO_4_ 10 g/L, MgSO_4_·7H_2_O 1 g/L and FeSO_4_·7H_2_O 1 g/L (pH 7.0). The cultures were grown at 36 °C with an agitation of 170 rpm for 12 h. Cells were harvested and suspended in 20 mM Tris lysis buffer (pH 7.0) containing 0.3 g/100 ml lysozyme, 0.1 g/100 ml Triton X 100 and 1 mM PMSF and incubated for 1 h at 30 °C. The lysate was centrifuged at 10,000*g* for 20 min at 4 °C. The supernatant thus collected was used to determine intracellular α-galactosidase activity.

### Enzyme and total protein assay

α-Galactosidase activity was measured according to the method of Dey et al. (1993). A reaction mixture containing 550 µl of 20 mM Tris buffer (pH 7.0), 100 µl of enzyme preparation and 250 µl of 0.2 M *pNPGal* was incubated at 50 °C for 10 min and the reaction was stopped by addition of 0.2 mM Na_2_CO_3_ and read at 405 nm. The activity was also measured using 2 mM raffinose as natural substrate in a reaction mixture containing 20 mM Tris buffer (pH 7.0) and enzyme preparation. The resulting amount of reducing sugar was determined by addition of 3,5-dinitrosalicylate reagent (Miller 1959) and the colour read at 540 nm. The concentration of reducing sugar, i.e., galactose, was calculated using standard galactose curve.

One enzyme unit (U) of activity was defined as the amount of enzyme required to produce one µmol of *ρ*-nitrophenol or reducing sugars (galactose) per min under the above mentioned assay conditions.

The protein content of the enzyme preparation was determined according to the method of Lowry et al. (1951) using Bovine Serum Albumin (BSA) as standard.

## Purification and characterization of enzyme α-galactosidase

### Step 1: ammonium sulphate precipitation

The precipitate obtained at 60 % (NH_4_)_2_SO_4_ saturation was harvested by centrifugation at 10,000*g* for 30 min, 4 °C. The pellet obtained was dissolved in 20 mM Tris–HCl buffer (pH 7.2) and dialysed at 4 °C with three changes of buffer at 4 °C.

### Step 2: ion exchange chromatography

The dialyzed sample was loaded on to Q-Sepharose fast flow column with a bed height of 9 cm and internal diameter of 0.5 cm which was previously equilibrated with 20 mM Tris–HCl, pH 7.2 and a flow rate of 1 ml/min. The column was washed with three bed volumes of 20 mM Tris–HCl, pH 7.2 to remove unbound protein. The bound proteins were eluted using NaCl gradient from 0.1 to 1 M. Both unbound and bound fractions were concentrated and analysed for α-galactosidase activity using ρNPGal as substrate.

### Step3: gel filtration chromatography

The fractions showing α-galactosidase activity were concentrated and loaded on to Sephacryl S-300 gel filtration column (1 × 90 cm) pre-equilibrated with 20 mM Tris–HCl, pH 7.2. 1 ml fractions were collected at a flow rate of 0.5 ml/min. Fractions exhibiting activity were concentrated and analysed for purity by PAGE.

### Native and SDS-PAGE electrophoresis

SDS Polyacrylamide gel electrophoresis was performed in 10 % gels to determine molecular mass and purity of the enzyme (Laemmli 1970).

The enzyme was also electrophoresed in Native 10 % gels to check for purity (Davis 1964). The protein bands were visualised by staining with Coomassie Brilliant Blue 250.

### pH optimum

The pH optima for activity of the enzyme was assessed by monitoring the enzyme activity in different buffers with pH ranging from 5.0 to 11.0—citrate phosphate buffer (pH 5.0–7.0), Tris–HCl buffer (pH 7.0–9.0) and glycine–NaOH buffer (pH 9.2–11.0). The pH stability of the purified enzymes Ag-I & II was analysed by incubating them in different buffers with pH values ranging from 5 to 11 for a time period of 1 to 24 h at 36 °C.

### Temperature optimum, stability and half-life

The temperature optima for maximal activity of the pure enzyme was determined by incubating the reaction mixture at different temperatures ranging from 30 to 90 °C in Tris–HCl buffer (pH 7.0). The half-life (*t*_1/2_ min) of the enzyme Ag-I was determined by incubating the enzyme at different temperatures ranging from 70 to 90 °C for 5 h.

### Determination of kinetic constants

The kinetic parameters, *K*_m_, *V*_max_ and *K*_cat_ values of the purified α-galactosidase enzyme Ag-I and Ag-II were determined from Lineweaver–Burk plot. The rate of hydrolysis was determined with concentration ranging from 0.4 to 2.8 mM for both the substrates—*p*-nitrophenyl-α-d-galactopyranoside (*p*NPGal) and raffinose against enzyme activity. The reaction was carried out at 50 °C in 20 mM tris buffer, pH 7.0. A graph was plotted with 1/v versus 1/s. *K*_m_ and *V*_max_ values were calculated based on linear regression slope. Further *K*_cat_ value was calculated using formula

1 $$ k_{\text{cat}} = V_{\hbox{max} } /E_{\text{t}} , $$

where *E*_t_ is the total enzyme concentration.

### Influence of metal ions, inhibitors and surfactants on α-galactosidase activity

The influence of different metal ions such as Mg^2+^, K^+^, Ca^2+^, Cu^2+^, Co^2+^, Ag^2+^, Hg^+^, Zn^2+^ and chelator like EDTA were studied by preincubating the enzymes Ag-I & II with 5 mM concentration of ions in 20 mM Tris buffer at pH 7.0 for 1 h at 36 °C and then checking for enzyme activity.

The activity was also monitored after pre incubation with sugars like glucose, galactose, lactose, stachyose and other inhibitors like urea, PCMB (*ρ*-Chloro-mercuric benzoic acid), β-mercaptoethanol and EDTA at a concentration of 5 mM for 1 h at 36 °C and also with detergents like SDS, Triton X-100 and Tween-20 at a concentration of 1 % for 4 h.

## Results and discussion

### Identification of the isolate

A microorganism with the ability to produce alpha galactosidase enzyme was isolated from sugarcane waste and was identified as Acinetobacter sp., from its morphological features, biochemical characteristics and 16srRNA sequencing analysis. Industrially important enzymes-pectinases, lipases and biodegradable enzymes were reported to be synthesised by Acinetobacter species (Shafqat et al. 2015; Das and Chandran 2011).

### α-Galactosidase purification and molecular weight

**Fig. 1:**
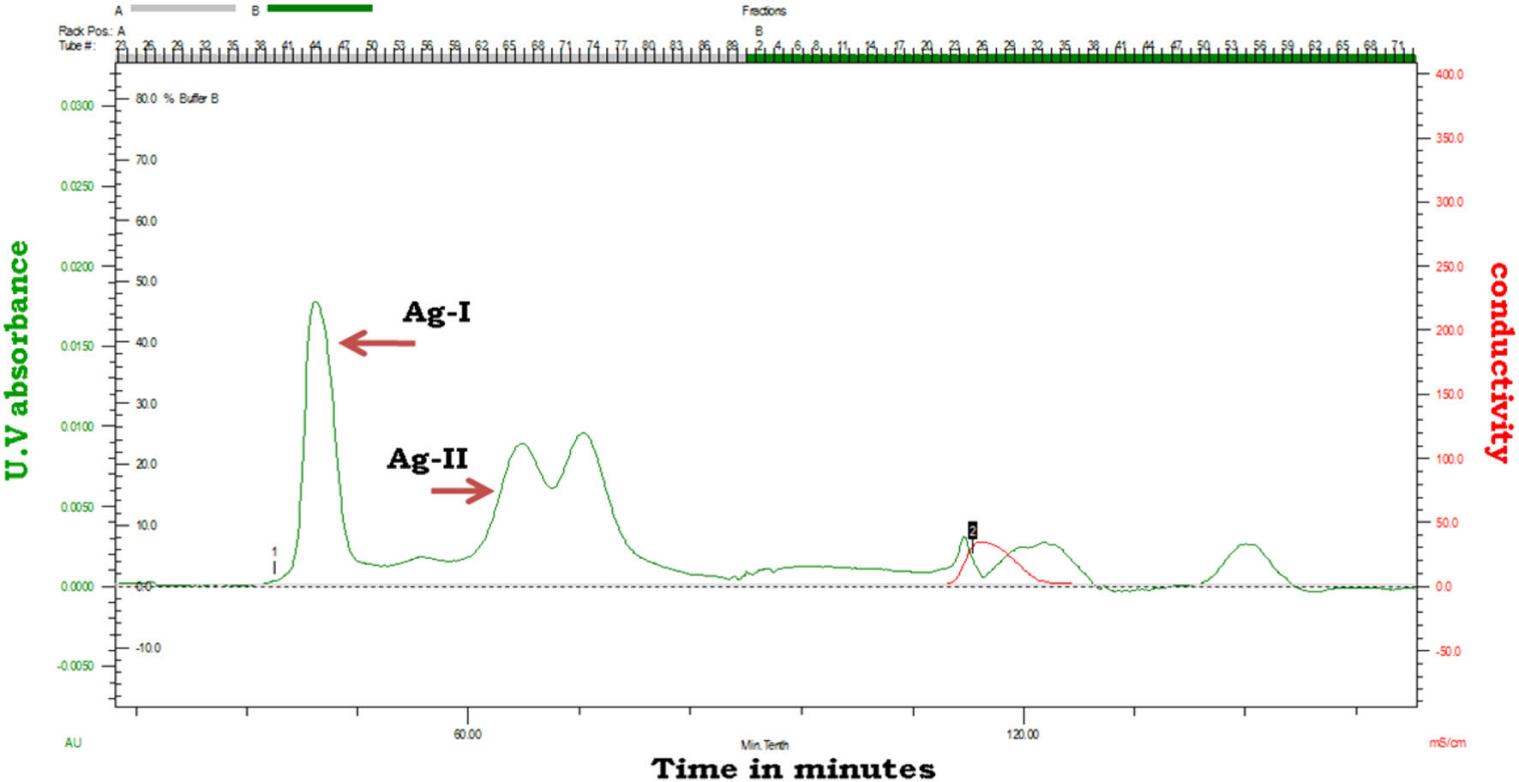
Gel-filtration chromatogram of *Acinetobacter* sp. multiforms. *Peak 1* showing higher activity was labelled as Ag I and *peak 2* showing lower activity was labelled as Ag-II

**Fig. 2:**
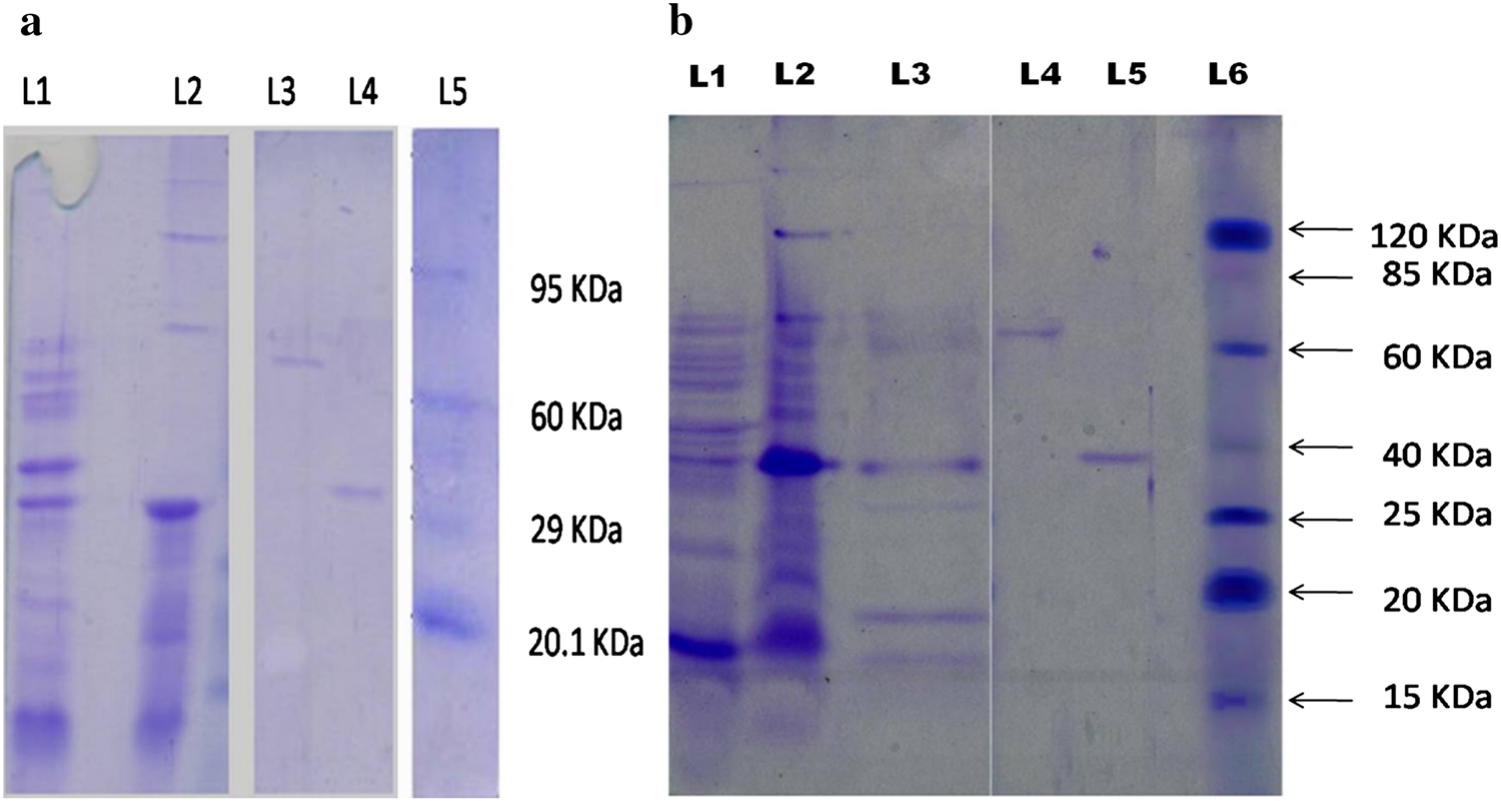
**a**, **b** Native and SDS PAGE analysis of purified alpha-galactosidase enzymes. **a** Native PAGE analysis with L1-Crude, L2-Ion exchange chromatography, L3-gel filtration, Ag-I, L4-gel filtration, Ag-II and L5-protein marker. **b** SDS PAGE analysis of purified alpha-galactosidase enzymes. *Lane 1* Crude, *lane 2* ammonium sulphate precipitation, *lane 3* ion exchange chromatography, *lane 4* gel filtration Ag-I, *lane 5* gel filtration Ag-II and *lane 6* prestained Benchmark

The enzyme was purified by (NH_4_)_2_SO_4_ precipitation, ion exchange chromatography and gel-filtration. Two forms of α-galactosidases were resolved in gel filtration chromatography with molecular weights of 65 kDa for Ag-I and 37 kDa for Ag-II (Fig. 1). Both the enzymes were monomeric (Fig. 2a, b). The enzyme recovery from the crude is shown in Table 1. Multiple forms of α-galactosidase have been reported in both prokaryotes and eukaryotes (Pederson and Goodman 1980; Gherardini et al. 1985; Alani et al. 1989).

**Table 1:** Summary of purification studies of alpha-galactosidases isolated from *Acinetobacter* sp.

Purification step	Total protein (mg)	Total activity (U ml^−1^)	Specific activity (U mg^−1^)	Recovery (%)	Purification fold
Crude	186	315	1.7	100	1
(NH_4_)_2_SO_4_ precipitation	32	165	5.1	52.3	3
Ion exchange	7.8	71.4	9.2	22.6	5.4
Gel filtration					
Ag-I	2.68	32.4	12.0	10.2	7.0
Ag-II	1.52	12.2	8.0	3.8	4.7

### pH optimum and stability

**Fig. 3:**
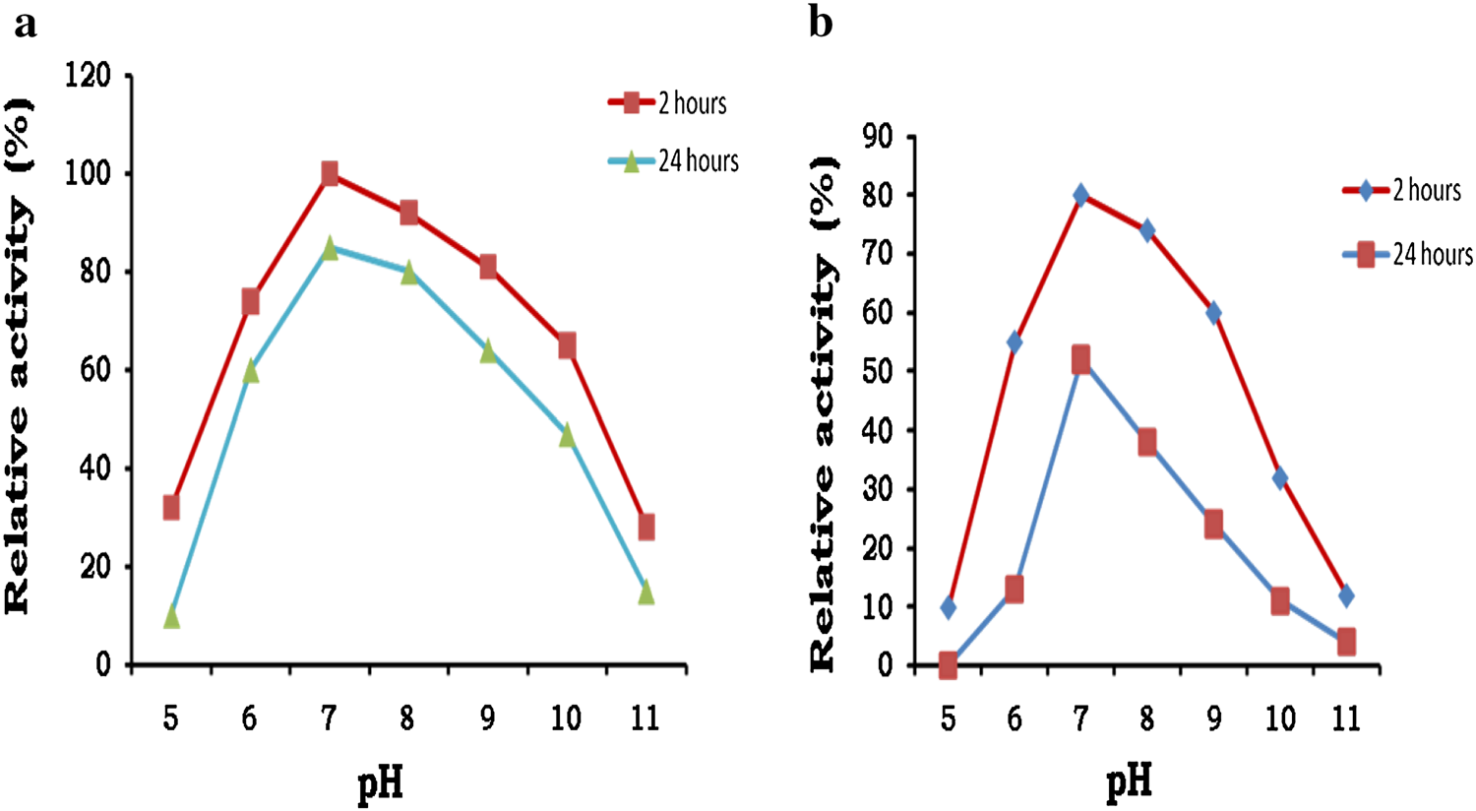
pH optimium of Ag-I (**a**) and Ag-II (**b**) after 2 and 24 h at 36 °C. pH optima was measured by incubating the enzyme with the substrate at different buffers over a pH range of 5.0 to 11.0

Both Ag-I and Ag-II were found optimally active and stable at neutral pH. Enzyme Ag-I was highly active and stable over broad pH range showing more than 60 % activity up to pH 9.0 with low activities at acidic pH. After 24 h of incubation at 36 °C, Ag-I retained 85 % enzyme activity at pH 7.0, whereas Ag-II retained 52 % activity (Fig. 3a, b). Most of the thermostable α-galactosidases were reported to be stable at neutral pH (Pederson and Goodman 1980; Frank et al. 1985; Alani et al. 1989; Gote et al. 2006).

### Optimum temperature and thermal stability

**Fig. 4:**
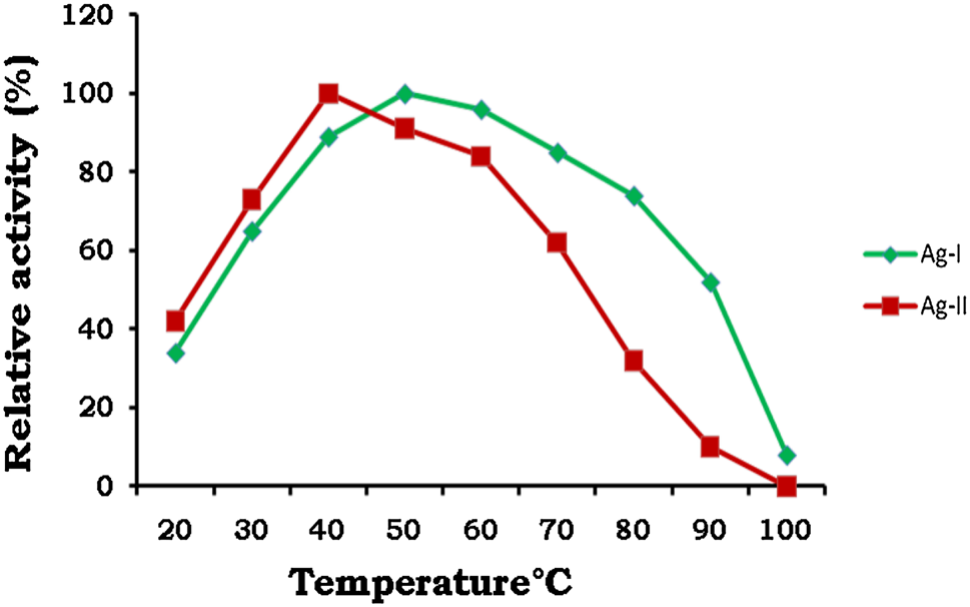
Temperature optima of purified enzymes Ag-I (*filled diamonds*) and Ag-II (*filled squares*)—temperatures ranging from 30 to 90 °C

**Fig. 5:**
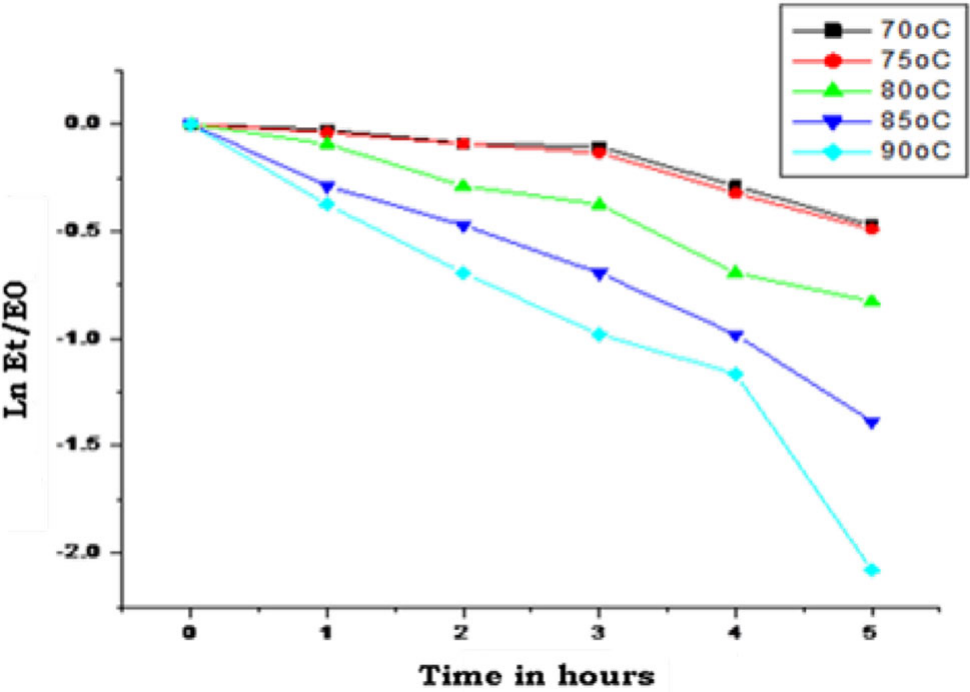
Thermal stability and half-life of Ag-I. The enzyme was incubated from 70 to 90 °C for 5 h. The half-life (*t*_1/2_ min) at each temperature was calculated from *kd* values obtained from a graph plotted in *E*_*t*_/*E*_0_ versus time in hours

The purified Ag-I enzyme showed an optimum temperature between 50 and 60 °C and was able to retain more than 80 % activity at 70 °C and thereafter a sharp decrease was observed at temperatures above 70 °C. Ag-II displayed an optimum temperature at 40 °C but markedly decreased at higher temperatures above 60 °C (Fig. 4). The half‐life of Ag‐I at 70 °C was 7.69 hours and thereby decreased rapidly (Fig. 5; Table 2). α-Galactosidases from *Bifidobacterium breve* (Xiao et al. 2000) and *Lactobacillus fermentum* (Carrera-Silva et al. 2006) were reported to have a temperature of 50 °C. α-Galactosidases isolated from few hyperthermophilic bacteria were reported to be stable with half-life ranging from 6 to 9 h at 85 °C (Miller et al. 2001; Duffaud et al. 1997) and 17 h at 80 °C (Giuseppin et al. 1993). Even in the present study the enzyme Ag-I had shown high thermal stability which can be further exploited for processing of legumes at higher temperatures.

**Table 2:** Half-life (*t*_1/2_) in hours calculated at temperatures 70–90 °C

Temperature (°C)	*kd* (moles)	Half-life, *t*_1/2_ (h)
70	0.09009	7.69
75	0.09546	7.26
80	0.17209	4.02
85	0.26377	2.62
90	0.37283	1.85

### Effect of metal ions on enzyme activity

**Fig. 6:**
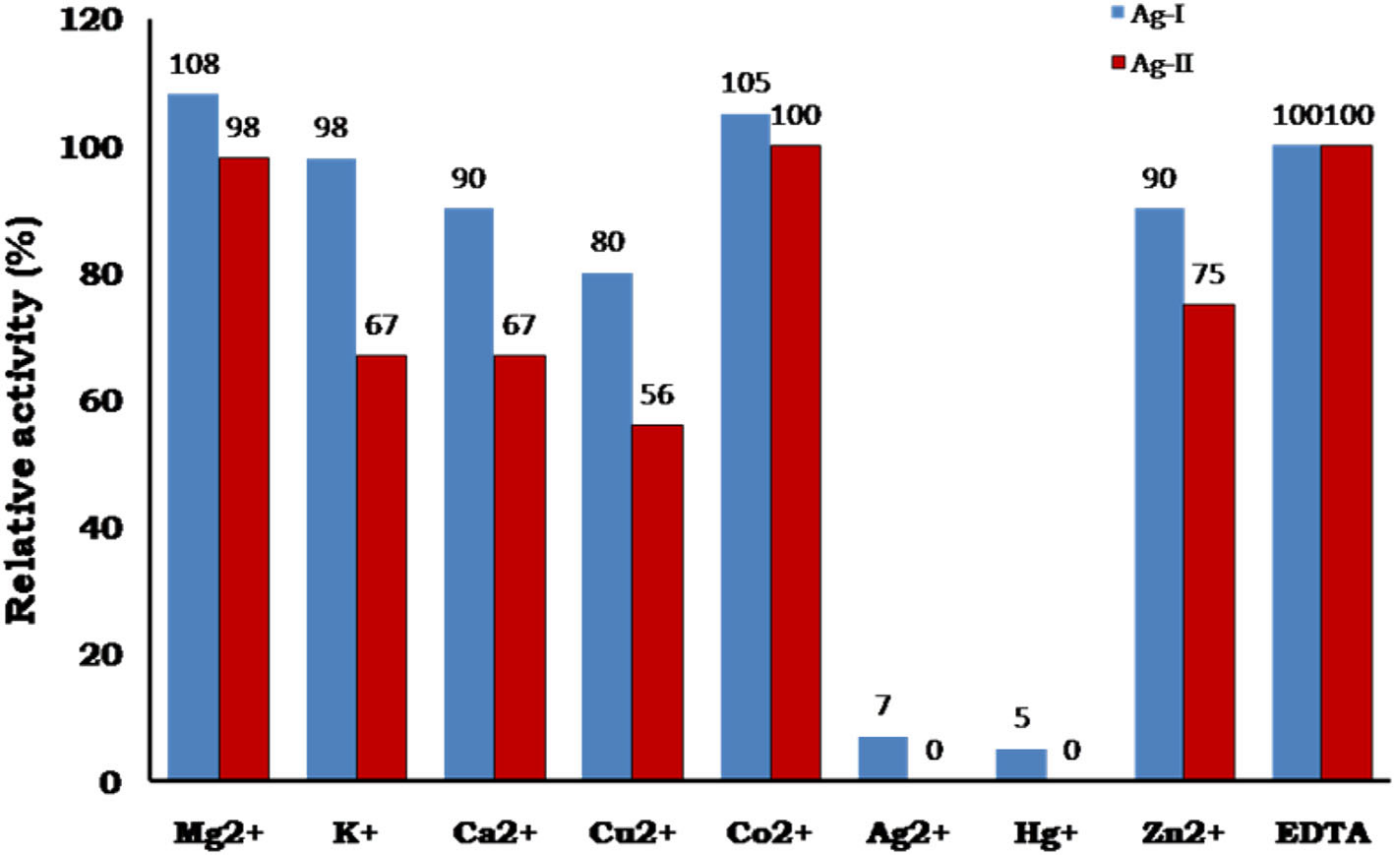
Effect of metal ions on purified enzymes Ag-I & Ag-II. The metal ions at a final concentration of 5 mM were preincubated with the purified enzyme in 20 mM tris buffer, pH 7.0 for 1 h at 36 °C

Assay of enzyme activity in the presence of different metal ions, sugar and non-sugar inhibitors and detergents indicated that both the enzymes (Ag I & II) were completely inhibited by Ag^2+^, Hg^+^ and partially in presence of Cu^2+^. Mg^2+^ and Co^2+^ stimulated activity whereas other metal ions showed greater than or equal to 90 % activity (Fig. 6). With respect to Ag II the activity was found to be reduced between 75 and 60 % in the presence of metal ions—K^+^, Ca^2+^ and Zn^2+^. Ag-I & II were neither not strongly stimulated by metal ions nor inhibited by EDTA, indicating that it does not require any cofactor for enzyme activity. α-Galactosidases isolated from different bacterial species were reported to be strongly inhibited by Ag^2+^, Hg^+^ ions and Cu^2+^ (Fridjonsson et al. 1999; Zhao et al. 2008; Akiba and Horikoshi 1976). Chinen et al. (1981) suggested that the inhibition may be due to reaction with amino, carboxyl and imidazolium group of histidine present at the enzyme active site.

### Effect of inhibitors and surfactants

The activity of enzymes (Ag-I & II) was decreased from 20 to 30 % in the presence of sugar inhibitors—glucose, galactose, lactose and stachyose (Table 3). The observed decrease in activity may be due to competitive inhibition by sugars (Zapater et al. 1990). Similar observation of substrate/product inhibition by various sugars was reported in other α-galactosidases from bacterial and fungal species (Gote et al. 2006; Falkoski et al. 2006). Triton X-100 and Tween-20 enhanced the activity of Ag I. A slight decrease in activity was observed in the presence of Beta-mercaptoethanol. Both the enzymes were also strongly inhibited by pCMB, a thiol modifier and SDS. Inhibition by pCMB may be due to reaction with thiol groups at active site (Dey and Pridham 1972; Gote et al. 2006) and that of SDS may be due to strong affinity to proteins and its denaturing property (Falkoski et al. 2006).

**Table 3:** Effect of inhibitors on purified enzymes, Ag-I and Ag-II

Inhibitors	Relative activity (%)	
	Ag-I	Ag-II
Control	100	100
Sugar inhibitors (5 mM)		
Glucose	82	75
Galactose	92	72
Lactose	74	70
Stachyose	71	63
Non-sugars (5 mM)		
PCMB	0	0
Urea	100	92
Beta-mercaptoethanol	88	74
EDTA	100	100
Surfactants (1 %)		
SDS	0	0
Triton X-100	110	100
Tween-20	128	100

Sugar and non-sugar inhibitors are incubated with purified enzymes at a concentration of 5 mM for 1 h at 36 °C. Detergents are incubated at a concentration of 1 % for 4 h

### Determination of kinetic constants for soluble enzyme Ag-I & II

The kinetic constants for both the forms of α-galactosidases are shown in Table 4. Both enzymes Ag-I and Ag-II have a higher affinity to synthetic substrate *pNPGal* (low *K*_m_ value) as compared with natural substrate raffinose. The rate of hydrolysis is high for raffinose (Ag-I) although the *K*_m_ value is high compared to synthetic substrate indicating that although the affinity is low, the catalytic processing is higher for the substrates.

**Table 4:** Kinetic constants of purified soluble enzymes Ag-I and Ag-II with natural substrate, raffinose and synthetic substrate pNPGal

Substrates	Maximum velocity (*V*_max_) µM min^−1^ ml^−1^	Michaelis constant (*K*_m_) mM ml^−1^	Turnover number (*K*_cat_) min^−1^	Specificity constant (*K*_cat_/*K*_m_) mM^−1^ min^−1^
Alpha-galactosidase I (Ag-I)				
ρNPGal	14.51	0.33	4.45 × 10^2^	13.48 × 10^2^
Raffinose	27.24	3.26	1.1 × 10^2^	0.33 × 10^2^
Alpha-galactosidase II (Ag-II)				
ρNPGal	6.73	0.68	1.52 × 10^2^	2.23 × 10^2^
Raffinose	5.44	0.74	0.6 × 10^2^	0.81 × 10^2^

## Conclusion

Multiple forms of intracellular α-galactosidases (Ag I & II) isolated from *Acinetobacter* sp. were purified to homogeneity. Of both the enzymes, Ag I was found to be thermostable up to 70 °C. This enzyme has potential applications of these enzyme preparations in high temperature catalytic processes.

## Abbreviations

SDS: Sodium dodecyl sulphate
pNPGal: *p*-Nitrophenyl-α-d-galactopyranoside
pCMB: Para-chloromercuribenzoic acid
kDa: Kilo Dalton

## References

[CR1] Akiba T, Horikoshi K (1976). Properties of α-galactosidase of alkalophilic bacteria. Agric Biol Chem.

[CR2] Alani SR, Smith DM, Markakis P (1989). Alpha galactosidases of *Vigna unguiculata*. Phytochemistry (Oxford).

[CR3] Buchanan RE, Gibbons NE (1974). Bergey’s manual of determinative bacteriology.

[CR4] Carrera-Silva EA, Silvestroni A, LeBlanc JG, Piard JC, deGiori GS, Sesma F (2006). A thermostable alpha-galactosidase from *Lactobacillus fermentum* CRL722: genetic characterization and main properties. Curr Microbiol.

[CR5] Chen M, Mustapha A (2012). Survival of freeze-dried microcapsules of α-galactosidase producing probiotics in a soy bar matrix. Food Microbiol.

[CR6] Chinen I, Nakamura T, Fukuda N (1981). Purification and properties of α-galactosidase from immature stalks of *Saccharum officinarum* (sugar cane). J Biochem.

[CR7] Clarke JH, Davidson K, Rixon JE, Halstead JR, Fransen MP, Gilbert HJ, Hazlewood GP (2000). A comparison of enzyme-aided bleaching of softwood paper pulp using combinations of xylanase, mannanase and alpha-galactosidase. Appl Microbiol Biotechnol.

[CR8] Das N, Chandran P (2011). Microbial degradation of petroleum hydrocarbon contaminants: an overview. Biotechnol Res Int.

[CR9] Davis PJ (1964). Disc electrophoresis II. Methods and application to human serum protein. Ann N Y Acad Sci.

[CR10] del Campillo E, Shannon LM (1982). An α-galactosidase with hemagglutinin properties from soybean seeds. Plant Physiol.

[CR11] Desnick RJ (2004). Enzyme replacement therapy for Fabry disease: lessons from two alpha-galactosidase A orphan products and one FDA approval. Expert Opin Biol Ther.

[CR12] Dey PM, Pridham JB (1972). Biochemistry of α-galactosidases. Adv Enzymol Relat Areas Mol Biol.

[CR13] Dey PM, Patel S, Brownleader MD (1993). Induction of α-galactosidase in *Penicillium ochrochloron* by guar (*Cyamopsis tetragonobola*) gum. Biotechnol Appl Biochem.

[CR14] Duffaud GD, McCutchen CM, Leduc P, Parker KN, Kelly RM (1997). Purification and characterization of extremely thermostable β-mannase, β-mannosidase and α-galactosidase from the hyperthermophilic eubacterium *Thermotoga**neapolitana* 5068. Appl Environ Microbiol.

[CR15] Falkoski DL, Guimaraes VM, Callegari CM, Reis AP, De Barros EG, De Rezende ST (2006). Processing of soybean products by semipurified plant and microbial alpha-galactosidases. J Agric Food Chem.

[CR100] Frank G, Martin B, Abigail SA (1985). Purification and characterization of two
α-galactosidases associated with catabolism of guar Gum and other α-
galactosides by *Bacteroides ovatus*. J Bacteriol.

[CR16] Fridjonsson O, Watzlawick H, Gehweiler A, Mattes R (1999). Thermostable α-galactosidase from *Bacillus stearothermophilus* NUB 3621: cloning, sequencing and characterization. FEMS Microbiol Lett.

[CR17] Gao Z, Arthur AS (1999). A novel alkaline α-galactosidase from melon fruit with a substrate preference for raffinose. Plant Physiol.

[CR18] Gao HW, Li SB, Bao GQ, Tan YX, Wang YL, Zhang YP, Ji SP, Gong F (2011) Properties of a novel α-galactosidase from *B. fragilis* and its potential for human blood-type B to O conversion. Sci Sin Vitae 41(10):1030–1036

[CR19] Gherardini F, Babcock M, Salyers AA (1985). Purification and characterization of two α-galactosidases associated with catabolism of guar Gum and other α-galactosides by *Bacteroides ovatus*. J Bacteriol.

[CR20] Giuseppin ML, Almkerk JW, Heistek JC, Verrips CT (1993). Comparative study on the production of guar α-galactosidase by *Saccharomyces cerevisiae* SU50B and *Hansenula polymorpha* 8/2 in continuous cultures. Appl Environ Microbiol.

[CR21] Gote MM, Khan MI, Gokhale DV, Bastawde KB, Khire JM (2006). Purification, characterization and substrate specificity of thermostable alpha-galactosidase from Bacillus stearothermophilus (NCIM-5146). Process Biochem.

[CR22] Kato K, Ikami T, Kono H, Yamauchi R, Ueno Y (1982). Transferase action of alpha-galactosidase from tubers of *Stachys affinisi*. Agric Biol Chem.

[CR23] Laemmli UK (1970). Cleavage of structural proteins during assembly of head of bacteriophage T4. Nature.

[CR24] Liu X, Champagne CP, Lee BH, Boye JI, Casgrain M (2014) Thermostability of probiotics and their α-galactosidases and the potential for bean products. Biotechnol Res Int 2014:21 pp 10.1155/2014/472723 PMC3948641 24744923

[CR25] Lowry OH, Rosebrough NJ, Farr AL, Randall RJ (1951). Protein measurement with the Folin phenol reagent. J Biol Chem.

[CR26] Miller GL (1959). Use of dinitrosalicyclic acid reagent for determination of reducing sugar. Anal Chem.

[CR27] Miller ES, Parker KN, Liebl W, Lam D, Callem W, Snead MA, Mathur EJ, Short JM, Kelly RM (2001) α-Galactosidases from *Thermotoga* species. Methods Enzymol 330:246–260 10.1016/s0076-6879(01)30380-4 11210503

[CR101] Naumoff DG (2001) Sequence analysis of glycosyl hydrolases: 13-fructosidase and agalactosidase superfamiJies. GlycoconjugateJ 18: 109

[CR102] Naumoff DG (2004) The α-galactosidase superfamily: Sequence based classification of α-galactosidases and related glycosidases. In: Proc. Fourth Int. Conf. on Bioinformatics of Genome Regulation and Structure, pp 25–30

[CR28] Patil AG, Praveen Kumar SK, Mulimani VH, Veeranagouda Y, Lee K (2010). α-Galactosidase from *Bacillus megaterium* VHM1 and its application in removal of flatulence-causing factors from soymilk. J Microbiol Biotechnol.

[CR29] Pederson DM, Goodman RE (1980). Isozymes of alpha-galactosidase from *Bacillus stearothermophilus*. Can J Microbiol.

[CR30] Puchart V, Vrsanska M, Bhat MK, Biely P (2001). Purification and characterization of alpha-galactosidase from a thermophilic fungus *Thermomyces lanuginosus*. Biochim Biophys Acta.

[CR31] Saishin N, Ueta M, Wada A, Yamamoto I (2010) Purification and characterization of α-galactosidase I from Bifidobacterium longum subsp. longum JCM 7052. J Biol Macromol 10(1):13–22

[CR32] Shafqat I, Shahzad S, Yasmin A (2015). Microbial potential of lipase production from different industrial effluents. J Appl Biol Sci.

[CR33] Singh N, Kayastha AM (2012). Purification and characterization of α-galactosidase from white chickpea (*Cicer arietinum*). J Agric Food Chem.

[CR34] Tsuboi K, Yamamoto H (2012). Clinical observation of patients with Fabry disease after switching from agalsidase beta (Fabrazyme) to agalsidase alfa (Replagal). Genet Med.

[CR35] Xiao M, Tanaka K, Qian XM, Yamamoto K, Kumagai H (2000). High yield production and characterization of α-galactosidase from *Bifidobacterium breve* grown on raffinose. Biotechnol Lett.

[CR36] Yamane T (1971) Decomposition of raffinose by α-galactosidase. An enzymatic reaction applied in the factory-process in Japanese beet sugar factories Sucr Belge Sugar Ind Abstr 90:345-348

[CR37] Zapater IG, Ullah AH, Wodzinski RJ (1990). Extracellular α-galactosidase from *Aspergillus ficuum* NRRL3135 purification and characterization. Prep Biochem.

[CR38] Zeyland J, Gawronska B, Juzwa W, Jura J, Nowak A, Slomski R, Smorag Z, Szalata M, Wozniak A, Lipinski D (2013). Transgenic pigs designed to express human α-galactosidase to avoid humoral xenograft rejection. J Appl Genet.

[CR39] Zhao H, Lu L, Xiao M, Wang Q, Lu Y, Liu C, Wang P, Kumagai H, Yamamoto K (2008). Cloning and characterization of a novel alphagalactosidase from *Bifidobacterium breve* 203 capable of synthesizing gal-alpha-1,4 linkage. FEMS Microbiol Lett.

